# The Prevalence of TEM and SHV Genes among Extended-Spectrum Beta-Lactamases Producing *Escherichia Coli* and *Klebsiella Pneumoniae*

**Published:** 2012

**Authors:** Fatemeh Riyahi Zaniani, Zahra Meshkat, Mahboubeh Naderi Nasab, Mehrangiz Khaje-Karamadini, Kiarash Ghazvini, Abdolrahim Rezaee, Habibollah Esmaily, Maryam Sadat Nabavinia, Mahboubeh Darban Hoseini

**Affiliations:** 1 *Microbiology and Virology Research Centre, Mashhad University of Medical Sciences, Mashhad, Iran*; 2 *Women's Health Research Centre, Mashhad University of Medical Sciences, Mashhad, Iran*; 3 *Department of Biostatistics in Health Sciences, Mashhad University of Medical Sciences, Mashhad, Iran*

**Keywords:** Escherichia coli, Extended spectrum beta-lactamase (ESBL), Klebsiella pneumonia, PCR, Prevalence, SHV gene, TEM gene

## Abstract

**Objective(s):**

Production of extended-spectrum beta-lactamases (ESBLs) by enteric bacteria continues to be a major problem in hospitals and community. ESBLs producing bacteria cause many serious infections including urinary tract infections, peritonitis, cholangitis and intra-abdominal abscess. The aim of this study was to determine the prevalence of ESBLs producing *Escherichia coli* and *Klebsiella pneumoniae* bacteria isolated from clinical samples of patients attending Imam Reza and Ghaem University Hospitals, Mashhad, Northeast of Iran.

**Materials and Methods:**

During 2009 and 2010, 82 strains of *E. coli* and 78 strains of *K. pneumoniae* were isolated from out-patients and hospitalized patients and they were examined by Oxoid combination disk test and PCR methods.

**Results:**

We found that 43.9% of *E. coli* and 56.1% of *K. pneumoniae* produced ESBLs. The frequency of SHV and TEM among the ESBLs producing isolates were 14.4% and 20.6%, respectively. Ratios of ESBLs positive isolates from out-patients to hospitalized patients were 24/33.

**Conclusion:**

This study shows that the prevalence of ESBLs producing *E. coli* and *K. pneumoniae* is high in both study groups (out-patients and hospitalized patients). Therefore it seems that continuous surveillance is essential to monitor the ESBLs producing microorganisms in hospitals and community.

## Introduction

Beta-lactam antibiotics are the most common drugs of any application in the treatment of bacterial infections (1). The beta-lactam antibiotics have been widely used since 1980 for the treatment of serious infections caused by gram-negative bacteria, but resistance against these antibiotic groups occurred quickly worldwide (2, 3).

Production of beta-lactamase enzymes is the main mechanism of bacterial resistance against various antibiotics of this class (4). More than 200 different types of extended spectrum beta-lactamases (ESBLs) have been reported around the world so far; they were often identified in Enterobacteriaceae family. *Klebsiella pneumoniae* is the most common bacterial production of ESBLs; similarly,* Escherichia coli* is the other very important microorganism. ESBLs productions are rarely observed among other bacteria (2, 3).

ESBLs are often plasmid mediated and most of the enzymes are members of TEM and SHV families (5, 6) that have been described in many countries (2, 3,7,8). The TEM was first reported in *E. coli* isolated from a patient named Temoniera in (9). The name of the other beta-lactamase, SHV, is due to sulf-hydryl variable active site (9).

ESBLs producing bacteria have been reported increasingly worldwide. They cause many clinical diseases including urinary tract infections, peritonitis, cholangitis and intra-abdominal abscess (10-12). Treatment of the infections caused by these organisms is a major challenge for health care facilities and preventive strategies. Since ESBLs are identified most commonly in *E. coli *and *K. pneumoniae,* we studied the prevalence of ESBLs producing in these bacteria isolated from out-patients and hospitalized patients at Imam Reza and Ghaem University Hospitals in Mashhad, Northeast of Iran. 

## Materials and Methods

This study was conducted at Mashhad University of Medical Sciences, Northeast of Mashhad, Iran. Totally, 160 isolates of *E. coli *and *K. pneumoniae* bacteria were selected from out-patients and hospitalized patients of Imam Reza and Ghaem University Hospitals during 2009 and 2010. Different clinical samples including urine, blood, wound and abscess aspirates, peritonitis and pulmonary secretions were processed in this study.

***Phenotypic confirmatory test (PCT)***

For ESBL assay, bacterial suspensions with concentration of 1.5×10^8^ cfu/ml (0.5 McFarland standard) were prepared in nutrient broth. Oxoid combination disk method was used for detection of ESBLs producing organisms. In this method the bacteria were cultured on a Muller-Hinton agar plate, then cefotaxim (30 µg), cefotaxim/clavulanate (10 µg), ceftazidime (30 µg) and ceftazidime/clavulanate (10 µg) disks (Mast, UK) were placed on media in 20-30 mm with other disks. The plates were incubated for 18-24 hrs at 37 °C. ESBLs producing organisms were detected by an at least 5 mm increasing of zone around cefotaxim/clavulanate and ceftazidime/clavulanate at least 5 mm.

***DNA extraction, PCR and sequencing***

The colonies of ESBLs producing organisms were suspended in TE buffer and their DNA were extracted by simple boiling (13). 

The PCR method for detection of SHV and TEM genes was performed as described previously with minor modifications (14-15); briefly, specific primers for the genes (forward primer 5´-TCAGCGAAAAACACCTTG -3´; Reverse primer 5´-CCCGCAGATAAATCACCA -3´ for SHV gene and forward primer 5´-GAGTATTCAACATTTCCGTGTC -3´; Reverse primer 5´-TAATCAGTGAGGCACCTATCTC -3´ for TEM gene) were used for PCR amplification that produced 471 bp and 861 bp PCR products for SHV and TEM genes, respectively. The PCR mixture consisted of 10 pmol of each primers, 1 μl DNA sample (3 μg/μl), 1.5 mM MgCl_2_, 0.2 mM each dNTP, and 5 u Taq DNA polymerase (Cinagen, Iran) in a total number of 50 μl of PCR reaction. Amplification of TEM and SHV genes was performed by following program: initial denaturation at 94 °C for 2 min and 35 cycles of 1 min at 94 °C, 30 sec at 52 °C and 1 min at 72 °C. Five min at 72 °C was considered for the final extension. Then, PCR products were analyzed by agarose gels electrophoresis. Eight PCR products from different types of the samples were sent for sequencing to Microgene Company, .

## Results

**Figure 1:**
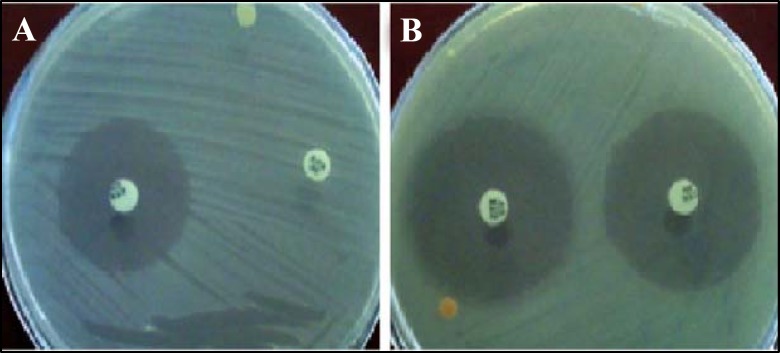
Oxoid combination disk method results. The ESBLs producing (A) and not producing (B) organisms were differentiated byta increasing of growth inhibition zone only around the cefotaxim/clavulanate (or ceftazidime/clavulanate) for at least 5 mm in ESBLs producing ones

Among all the samples, urine samples were the most positive ones (n= 133, 1.83%). In this study, a total number of 160 bacteria isolates (*E. coli* n=82, *K. pneumoni*ae n= 78) were collected; of these 57 isolates (35.6%) were ESBLs producing organisms (Fig 1, and Table 1). Our results showed that 25 (15.62%) of isolated *E. coli* and 32 (20%) of isolated *K. pneumoniae* were ESBLs producing organisms.

**Table 1:** The prevalence of ESBLs producing *Escherichia coli* and *Klebsiella pneumoniae* by disk diffusion method

Bacteria	ESBLs - positive		ESBLs-negative		total	
	Number	%	Number	%	Number	%
*E. coli*	25	15.625	57	35.625	82	51.25
*K. pneumoniae*	32	20	46	28.75	78	48.75
Total	57	35.625	103	64.375	160	100

**Table 2:** Prevalence of ESBLs producing bacteria by disk diffusion method in out- patients and hospitalized patients

Patients	ESBLs- positive		ESBLs-negative		Total	
	Number	%	Number	%	Number	%
Hospitalized patients	33	57.897	47	45.63	80	50
Out-patients	24	42.103	56	54.37	80	50
Total	57	100	103	100	160	100

**Figure 2:**
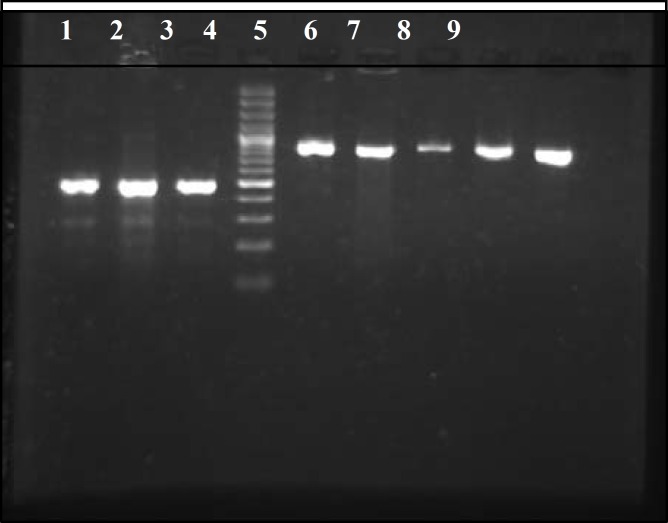
PCR results for SHV and TEM genes. Lane numbers 1, 2 and 3 are showed a 471 bp fragment of SHV gene. Lane numbers 5-9 are showed a 861 bp fragment of TEM gene and lane 4 is showed the 100 bp DNA size marker

**Table 3:** The prevalence of TEM and SHV genes in ESBLs producing organisms in hospitalized patients and out-patients

	*Klebsiella pneumoniae*				*Escherichia coli*				Total
	TEM positive	SHV positive	TEM and SHV positive	TEM and SHV negative	TEM positive	SHV positive	TEM and SHV positive	TEM and SHV negative	
Hospitalized patients	5	0	0	9	2	4	11	2	33
Out-patients	6	0	0	5	3	2	6	2	24
Total	11	0	0	14	5	6	17	4	57

The distribution of ESBLs producing organisms based on out-patients and hospitalized patients are shown in Table 2. The obtained data showed the rate of ESBLs producing organisms in hospitalized patients were higher than out-patients but their differences was not statistically significant (*P*-value= 0.137).

In PCR method the distribution of TEM and SHV genes in isolated ESBLs producing organisms were 33 (20.6%) and 24 (14.4%), respectively (Figure 2, Table 3).

Eight PCR products from different kinds of samples were** s**equenced and reported as Iranian strains in Gen Bank (Accession Numbers: GU338982, GU338983, GU338984, GU338985, GU338986, GU338979, GU338980 and GU338981). The results obtained from sequencing was compared with the published sequences in Gen Bank and it showed that there was 99-100% (for SHV beta-lactamase gene) and 100% (for TEM-1b beta-lactamase gene) homology between our PCR products and those mentioned in Gen Bank.

## Discussion

Resistance to beta-lactam antibiotics of Gram- negative bacteria isolated from clinical samples has been increased worldwide (16). The aim of this study was to evaluate the prevalence of ESBL producing *E. coli* and *K. pneumoniae* and to detect the presence of TEM and SHV genes among ESBL producing isolates. Based on the results of this study, the prevalence of ESBL producing *E. coli* and *K. pneumoniae* was high (15.62% and 20%, respectively) and a total number of about 68.5% of ESBL producing isolated bacteria were TEM and/or SHV positive. A number of previous studies have showed the high prevalence of ESBLs producing *E. coli* and *K. pneumoniae*. In , the prevalence of ESBLs producing *E. coli* and *K. pneumoniae *isolates varies in different countries. In , the prevalence of the organisms was reported 46.51% in *E. coli* and 44.44% in *K. pneumoniae* isolates (17). The prevalence of the organisms in was in the range of 8.5% to 29.8% in *K. pneumoniae* and 1.5% to 16.7% in* E. coli* (18). In a study in , the prevalence of the ESBLs producing organisms in children was reported 27% in *E. coli* and 64% in *K. pneumoniae* isolates (19). In Korea, the prevalence of these organisms was in the range of 4.8%-7.5% and 22.5%-22.8% for *E. coli* and *K. pneumoniae*, respectively (20). In , the prevalence of the ESBLs producing *E. coli* and *K. pneumoniae* was reported 41% in *E. coli* and 36% in *K. pneumoniae* isolates (21). In another study in , the prevalence of the ESBLs producing *E. coli* isolates was reported 56.9% (22).

Reported ESBLs producing rates in *E. coli* and *K. pneumoniae* isolates from various parts of Iran varies from 8.9% to 67% in* E. coli* and 20.3% to 52% in *K. pneumoniae* isolates (5, 23-27). In our study, the prevalence of ESBLs producing *E. coli* and *K. pneumoniae* was 15.6% and 20%, respectively.

 In a study that was carried out by Fazly Bazzaz *et al* in 2007 in Mashhad, the prevalence of ESBLs producing *E. coli* and *K. pneumoniae* was reported 57.5% and 61% and generally the prevalence of ESBLs producing organisms were 59.2% by Kirby-Baure disk diffusion method and the phenotypic disk confirmatory test. They also showed that the ratios of ESBLs producing isolates from hospitalized patients to out-patients were 94 to 28 (23). In our study, the prevalence of ESBLs producing organisms was lower in comparison with the reported prevalence by their study (23). Also the ratios of ESBLs producing organisms in hospitalized patients to out-patients were 57.9 to 42.1 that were different from a previous study which suggested that although most of the resistance to antibacterial agents is associated with admission to hospitals, ESBLs producing bacteria may have both community and hospital acquired sources. The ESBLs producing organisms was higher in *K. pneumoniae* compared to* E. coli* isolates in both studies.

In another study that was performed by Kalematizadeh in 2008 in , the prevalence of ESBLs *K. pneumoniae* was 43% (44% in hospitalized and 18% in out- patients) (7). In Isfahan, Dr Rastegar Lari *et al* showed 51% of isolated *E. coli* and 70% of isolated *K. pneumoniae* were ESBLs producing bacteria (28). Their results showed different prevalence of ESBLs producing bacteria compared with our finding. Therefore the pattern of ESBLs producing bacteria varies in different parts of Iran and separate studies of the ESBLs producing bacteria is necessary in various parts to estimate the antibiotic resistance correctly for taking steps for reducing these resistances.

Our molecular study revealed the ESBLs producing organisms contained TEM (20.6%), and SHV (14.4%) genes by PCR. The TEM gene has high frequency compared to SHV gene; a fact which is similar to previous studies (8, 29) but it was different compared to Taşli *et al* and Ramazanzadeh's results (30, 31). Also, in our study, the SHV gene was not found in ESBLs producing *E. coli* isolates. Therefore the distribution pattern of TEM and SHV genes in isolated *E. coli* and *K. pneumoniae* is also different in various parts of Iran.

In the Rastegar Lari study, ESBLs producing *E. coli* had 85.6% TEM, 69.2% SHV and 53.8% in both genes that was much higher than in our results (28). In another study in , the ESBLs producing organisms (*E. coli* and *K. pneumoniae*) were also positive for SHV (52.7%) and TEM (32.4%) genes (30); and the frequency of SHV gene was higher than TEM gene that was different from our results. Some ESBLs producing bacteria showed negative results in PCR method for SHV and TEM genes based on our study, therefore other beta-lactamases genes may be involved in ESBLs resistance. However, further studies are required for finding the other genes in ESBLs producing *E. coli* and K*. pneumoniae* bacteria.

## Conclusion

In short, the prevalence of ESBLs producing organisms in Mashhad is high. It seems necessary for clinicians and health care systems to be fully aware of ESBLs producing microorganisms. Also, the ESBLs production monitoring is recommended to avoid treatment failure and suitable infection control in Iran.
